# *Brazilian National Survey on Child Nutrition*: evidence for food and nutrition policies

**DOI:** 10.1590/0102-311XEN108923

**Published:** 2023-08-28

**Authors:** Gilberto Kac, Inês Rugani Ribeiro de Castro, Elisa Maria de Aquino Lacerda

**Affiliations:** 1 Instituto de Nutrição Josué de Castro, Universidade Federal do Rio de Janeiro, Rio de Janeiro, Brasil.; 2 Instituto de Nutrição, Universidade do Estado do Rio de Janeiro, Rio de Janeiro, Brasil.

Adequate nutrition during childhood represents the basis for child survival, growth, and development, with repercussions in the later stages of life. For this reason, it is a priority in the national and international agendas of public health and child protection policies and is among the objectives of the Sustainable Development Goals ^1^ ^,^ ^2^ ^,^ ^3^ ^,^ ^4^. However, globally, at least one in three children under 5 years old is affected by one or more forms of malnutrition - such as undernutrition, overweight, and micronutrient deficiencies. While the situation of child nutrition has improved worldwide, many children still lack adequate food and nutrition, especially those most vulnerable: the youngest, the poorest, and those affected by humanitarian crises ^5^.

The *Brazilian National Survey on Child Nutrition* (ENANI-2019) was conceived with the primary purpose of producing qualified scientific evidence to support the evaluation, formulation, and reorientation of Brazilian policies in the field of child nutrition. ENANI-2019 was funded by the Brazilian Ministry of Health via the CNPq/MS/SCTIE/DECIT/SAS/DAB/CGAN Grant Scheme n. 11/2017 and provided information after 13 years without national data on this topic - since the 2006 *Brazilian National Survey on Demography and Health of Women and Children* (PNDS 2006) ^6^. 

ENANI-2019 was structured in three axes - breastfeeding and food consumption, anthropometry, and micronutrients - and its objective was to evaluate breastfeeding practices, complementary feeding and food consumption, anthropometric nutritional status, and the epidemiology of micronutrient deficiencies among Brazilian children under 5 years old, according to the country’s macro-regions and age group, and to measure the inequalities in these indicators. The sample comprised 12,545 households and 14,558 children and their biological mothers living in 123 municipalities ^7^.

ENANI-2019 is the first Brazilian population-based and nationally representative study, in which children between 6 months and 5 years of age were measured for a broad spectrum of biochemical markers - such as hemoglobin, and vitamins B1 and B6 in whole blood and serum levels for C-reactive protein, retinol, ferritin, vitamins B12, D and E, folate, zinc, and selenium ^8^. It is also the first time that detailed data on infant feeding and breastfeeding-related practices, such as human milk donation and cross-feeding, have been collected in a sample of children with national representativeness ^7^ ^,^ ^9^.

Most of the children surveyed by ENANI-2019 were born during Brazil’s economic and political crisis and before the COVID-19 pandemic. Thus, ENANI-2019 details the profile of food and nutrition of Brazilian children in a context of dismantling of social policies and of pre-pandemic. With the intention of articulating with Brazilian public policies, one of the objectives of this *Suplement*, entitled *Brazilian National Survey on Child Nutrition: Evidence for Food and Nutrition Policies*, is to analyze the changes in this field within the perspective of the nutritional transition, comparing the most recent data with those of the PNDS 2006.

The set of materials presented in this *Supplement* - this editorial, a debate article commented by researchers and policy makers, 2 essays, an interview, a methodological article, 5 original articles, and a brief communication - represents a little of each of the three axes of ENANI-2019. It is with great joy that we present this publication of CSP.

The central text of this *Supplement* ^10^ deals with the nutritional transition in Brazilian children based on expanded data derived from PNDS 2006 and ENANI-2019. It exposes a reduction in the prevalence of anemia and vitamin A deficiency, as well as a reduction in regional inequalities, according to educational level and race/skin color for stunting, anemia, vitamin A deficiency, exclusive breastfeeding in children < 6 months of age, and continued breastfeeding in children aged 12-23 months of age. These changes can be attributed to improvements in living conditions and expansion of food and nutrition policies implemented before 2015. It is not possible to determine whether these indicators had better profiles in 2015 and deteriorated after that year, when economic austerity measures and a coordinated action to dismantle public policies that guaranteed rights was put into practice. On the other hand, it also an increase in the prevalence of excessive weight within the studied age group, suggesting the need to intensify public policies that promote and facilitate access to adequate and healthy food and measures that discourage the consumption of ultra-processed foods.

In the period between PNDS 2006 and ENANI-2019, stunting - an important indicator of childhood health - remained stable at 7% among children < 5 years old. However, this stability is not observed in the different age groups. Among children < 1 year of age, the prevalence of stunting increased from 4.7% to 9%, and we can assume that the main reason for its increase is the birth of these children in a context in which living conditions had deteriorated. This prevalence may be even higher in the future, if structural measures aimed at improving living conditions and protecting children are not intensified ^11^. 

The topic of the double burden of malnutrition was also addressed in this *Supplement* ^12^, considering that it is a global challenge on the rise. The main result revealed that overweight in the mother-child dyad and overweight in the mother and any type of malnutrition in the child (stunting and wasting or underweight) were the main expressions of malnutrition in households surveyed by ENANI-2019, affecting more than 1.8 million dyads. The prevalence of dyads with overweight mothers and children with stunting was higher in mothers with ≤ 7 years of schooling (4.8%) compared to those with ≥ 12 years (2.1%). When the results of the double burden of malnutrition were compared with those of the PNDS 2006, an increase from 2.7% to 5.2% was observed, showing the need to prioritize interventions for the most vulnerable groups, such as women who are older and with low education.

This *Supplement* highlights the article ^13^ that investigated the main factors associated with two outcomes of great relevance to public health, anemia and vitamin A deficiency, considering a hierarchical analysis and based on the theoretical model of United Nations Children’s Fund (UNICEF), which considers three levels of determination. Children living in the North Region had the highest prevalence of anemia, and those born in the Central-West, the highest prevalence of vitamin A deficiency. Low maternal schooling, maternal age < 20 years, brown skin color, and the existence of more than one resident < 5 years old in the household were factors associated with a prevalence of these outcomes. The results reinforce the importance of public policies focused on the most vulnerable groups in the various Brazilian regions.

Regarding dietary practices, two articles are presented. The first ^14^ describes the prevalence of minimum dietary diversity and of ultra-processed foods consumption in children 6-23 months of age according to socioeconomic variables. The prevalence of minimum dietary diversity was 63.4%, being lower in the North Region and for children with caregivers with ≤ 7 years of schooling. The prevalence of ultra-processed foods consumption was 80.5% and was higher in the North Region. The low dietary diversity and the high presence of ultra-processed foods in the diet of Brazilian children, especially in the most vulnerable groups, indicate the inadequacy of diets and the need to strengthen policies and programs to ensure an adequate and healthy child diet.

The second article ^15^ on feeding practices presents unprecedented data on the frequency of cross-feeding and donation and reception of milk via human milk banks. Cross-breastfeeding was practiced by 21% of mothers of children < 2 years old who breastfed at least once. The prevalence of breastfeeding another child was 15.6% and that of allowing their child to be breastfed by another woman was 11.2%. Almost 5% of the women donated milk to a human milk bank and 3.6% received donated milk. While cross-breastfeeding is not recommended by the Brazilian Ministry of Health due to the risk of transmission of HIV and other infectious agents, human milk donation is a highly recommended practice and has the potential to save lives. The debate on this topic is necessary and needs to be expanded. 

Few studies focus on the use of micronutrient supplements among children, especially at a national level. In this sense, the ENANI-2019 ^16^ data allowed us to characterize the micronutrient supplements intake in children 6-59 months of age according to macro-regions, caregiver education, type of service in which the supplement was prescribed, among others. The prevalence of intake of these products was 54.2% nationwide and reached 80% for children in the North Region. The prevalence of supplements containing only iron was 14.6% and containing only vitamin A, 23%. These results revealed that the coverage of the National Iron Supplementation Program was low and that a significant portion of supplement consumption occurs outside the programs of the Brazilian Ministry of Health. The indiscriminate consumption of micronutrient supplements is a problem for which health managers need to be more aware.

One of the contributions of ENANI-2019 was the update of the National Wealth Score (IEN), a synthetic index for assessing socioeconomic conditions at the household level, which was described in the methodological article that compose this *Supplement* ^17^. The IEN included items such as the education level of the mother or caregiver, the number of rooms and bathrooms in the home and the presence of television, car, radio, refrigerator or freezer, washing machine, microwave device, telephone, computers, air conditioning, mobile phone, internet service on the mobile phone, and internet at home. The principal components procedure was used in its estimation, being calculated with and without the sample design. The validation results revealed that the mean IEN score was lower in households with families participating in the Brazilian Income Transfer Program, in households with families with food insecurity, and in households with stunted children. These results indicate that the IEN presented an adequate performance in the evaluation of the socioeconomic conditions of households with children < 5 years old. 

In an essay ^18^ on the implementation of strategies and programs in the field of infant feeding and nutrition, the authors contextualize these programs and, through case studies, present two initiatives in the field of breastfeeding, focusing on the scope of the implementation analysis and the challenges encountered. This essay adds to the findings of ENANI-2019 and shows how the Implementation Science has been used to contribute to the achievement of adequate nutrition by 2030. 

Another essay ^19^ uses the ENANI-2019 data to reflect on the adequacy of the framework and classification system used to discuss all forms of malnutrition. The authors discuss the limitation of current approaches and the absence of approaches that consider the low quality of food. In this sense, the authors suggest an alternative approach focused on the classification of dietary patterns and their changes, rather than considering only health outcomes.

Finally, this *Supplement* brings an interview with Malaquias Batista Filho ^20^, Professor Emeritus of the Federal University of Pernambuco. Wise words from one of the most important scientist in the study of health and nutrition in Brazil. The interview addresses the professor’s experience in the field of food and child nutrition policies, the evolution of the nutritional epidemiological profile of Brazilian children, and the challenges of the current scenario of food and nutrition among this population group. 

This *Supplement* adds to the research reports published on the ENANI-2019 website (https://enani.nutricao.ufrj.br/index.php/relatorios/) and the methodological articles ^7^ ^,^ ^8^ ^,^ ^9^ ^,^ ^21^ ^,^ ^22^ published in the Thematic Section of CSP in August 2021, offering evidence and reflections on the contemporary context of child nutrition in Brazil and contributing to the advancement of knowledge production on this theme. With great enthusiasm, on behalf of the entire ENANI-2019 team and its collaborators, we invite you to enjoy this reading.
